# The role of air quality monitoring networks in supporting health research and legislation

**DOI:** 10.1186/1753-6561-7-S5-O11

**Published:** 2013-08-30

**Authors:** Harish Phuleria

**Affiliations:** 1 Exposures Scienses Group, SwissTPH, Basel, Switzerland

## 

Pollutants in the ambient air result in personal exposure, target tissue exposure and health responses. Ambient air quality measurement using various indicators is important in exposure assessment. Establishment of standard air quality monitoring networks helps in scientific investigation of health effects of air pollution, national and international reporting and to enforce regulations.

Particle mass concentrations (PM10, PM2.5) alone might not be sufficient to study particle related health effects. Additional health relevant particle data including ultra fine particles and/or specific toxic constituents are also needed. Quality assurance and control by regular calibrations and parallel measurements are of crucial importance for comparability of sites and establishment of trends. The SAPALDIA cohort study relies on the air quality monitoring network established in Switzerland for ambient air quality data.

Data from fixed site monitors and other sampling stations are used to create exposure models using dispersion, statistical and GIS parameters to arrive at individual exposure estimates. This air pollution exposure assessment aids epidemiological environmental health research. Evidence based on scientific research helps in the formulation of air quality regulations and standards. Good networks which provide consistent long term data from varied environments are needed with frequent collaborations among monitoring agencies and scientific disciplines.

